# Esophagitis after Clozapine Use in a 61-Year-Old Woman with Refractory Schizophrenia

**DOI:** 10.1155/2022/7033038

**Published:** 2022-06-03

**Authors:** Sung Min Ma, Junghyun Lim, Chunsu Jiang, Luminita Luca

**Affiliations:** ^1^Department of Psychiatry and Behavioral Sciences, University of Miami Leonard M. Miller School of Medicine, Miami, FL 33136, USA; ^2^Department of Medicine, University of Miami Leonard M. Miller School of Medicine, Miami, FL 33136, USA; ^3^Division of Gastroenterology, University of Miami Leonard M. Miller School of Medicine, Miami, FL 33136, USA

## Abstract

Clozapine-induced esophagitis has been rarely reported. We herein report a case of a 61-year-old woman with schizophrenia who developed hematemesis, fever, and tachycardia after the initiation of clozapine. An esophagogastroduodenoscopy showed esophageal mucosal ulcerations. Her gastrointestinal symptoms resolved with pantoprazole, allowing continuation of her clozapine treatment. We report here an unusual association of severe esophagitis with clozapine use.

## 1. Introduction

Clozapine is an antipsychotic medication often used in patients with treatment resistant-schizophrenia refractory to multiple antipsychotics. Like other atypical antipsychotic agents, clozapine acts as an antagonist not only on the dopamine type 2 (D2) receptor but also on the 5-hydroxytryptamine (serotonin) type 2A (5-HT2) receptor [1]. Moreover, clozapine has complex and unique binding properties with various other receptors (several subtypes of dopamine, serotonin, muscarinic, adrenergic, and glutamate receptors) [2, 3]. Although clozapine is widely considered the gold standard in the treatment-refractory schizophrenia, it is also known for its wide-ranging adverse effects. Its side effect profile includes the metabolic side effects with propensity for weight gain, agranulocytosis, seizure, and myocarditis [4]. Clozapine's highly antimuscarinic property is known to cause constipation and ileus, which in severe forms may lead to fecal impaction and colonic ischemia via fecal mucosa compression [5–7]. Due to the increasing number of reports that describe severe complications of untreated constipation with use of clozapine, the Food and Drug Administration (FDA) strengthened the warning about these potentially fatal side effects. Yet, clozapine's effect on the esophagus has been rarely described with few case reports [8, 9]. Here, we present a patient with schizophrenia who developed severe esophagitis upon initiation of clozapine.

## 2. Case Presentation

A 61-year-old Spanish-speaking woman with longstanding schizophrenia who recently immigrated from the Caribbean and Central America region presented to a psychiatric emergency room accompanied by her daughter due to aggressive behavior at home. In the emergency room, she exhibited religious and paranoid delusions and stated that she was the mother of Jesus Christ. She displayed agitated and aggressive behavior, as she attempted to move around and climb furniture. Physical exam was otherwise grossly unremarkable, including a soft and nontender abdomen. Laboratory tests showed electrolytes, thyroid-stimulating hormone level, and complete blood count that were within normal limits. Urine toxicology could not be done in the emergency room due to patient's disorganized behavior; however, per her family members, patient had not recently used alcohol or recreational drugs. She was previously stable on monthly haloperidol decanoate, which was no longer effective for the patient. She also had failed trials of olanzapine and risperidone. Per her caregiver, her symptoms deteriorated after immigrating to the United States two years prior to admission. She was admitted to an inpatient psychiatric unit. Spanish interpreter services were used to conduct the daily interviews. While inpatient, she received quetiapine (initially monotherapy, titrated up to 700 mg/day), then along with haloperidol (up to 10 mg/day), and divalproex sodium extended release (up to 500 mg/day). She continued to express religious delusions and visual and auditory hallucinations as she talked with angels. Due to persistence of psychosis, clozapine was introduced at 12.5 mg daily on day 25 and was steadily increased while other psychotropic medications were tapered down. To prevent constipation, she was started on docusate and polyethylene glycol. Meanwhile, she continued to express delusions of pregnancy, often pointing towards her abdomen. Due to disorganized thoughts, the review of system proved challenging.

**Figure 1:**
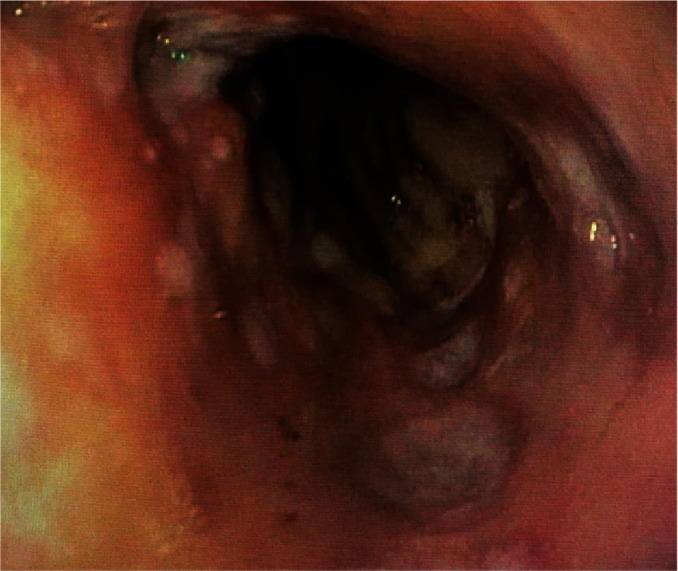
Endoscopic finding of the distal esophagus showing erythema and multiple discreet, punctate ulcerations.

On day 39, the patient developed coffee-ground emesis. Her psychotropic medications at the time were clozapine (total daily dose of 125 mg) and quetiapine (total daily dose of 150 mg). She was transferred to a medicine service, where she was febrile (38.9°C), tachycardic to 122 beats/minute, and normotensive. Physical examination revealed soft, nontender, and nondistended abdomen. Laboratory results were notable for leukocytosis (WBC: 12,900 cells/cmm). Hemoglobin level dropped to 10.8 g/dL from a baseline of 12 g/dL. She had a normal platelet count, an international normalized ratio of 1.1, a normal lactic acid level, and hypoalbuminemia (3.0 g/dL). An abdominal X-ray revealed significant stool burden. An esophagogastroduodenoscopy (EGD) showed mucosal changes characterized by erythema, erosion, friability, granularity, nodularity, and sloughing in the mid and distal esophagus (Figure 1), as well as gastroesophageal flap valve classified as Hill Grade III. Pathology was consistent with inflammation of the esophageal mucosa with necrotic features, with negative immunohistochemistry for CMV and fungal PAS stain. With conservative management with pantoprazole 40 mg twice a day, patient showed clinical improvement of gastrointestinal symptoms. She tolerated oral intake well with resolution of coffee-ground emesis and hemoglobin remained stable. Meanwhile, she was continued on clozapine 50 mg twice a day and transferred back to the psychiatry unit on day 48.

In the psychiatry unit, the patient continued to exhibit psychotic symptoms. She continued to exhibit delusions of pregnancy and was disrobing, saying that she was Eve (the biblical character). Decision was made to gradually increase clozapine under close monitoring. On total daily dose of 350 mg, clozapine and norclozapine levels were 560 mcg/L (close to the upper limit of therapeutic range) and 177 mcg/L, respectively. She also received divalproex sodium 750 mg as an adjunctive therapy and lorazepam to treat agitation. She was continued with pantoprazole and bowel regimen with docusate and magnesium hydroxide. She had no further episodes of coffee-ground emesis or other symptoms concerning for persistent esophagitis. There was improvement in her agitation but she continued to exhibit religious delusions, stating that she was talking to angels. Patient was subsequently transferred to a long-term treatment facility with a plan to repeat EGD in 2 months.

## 3. Discussion

Clozapine-induced esophagitis has been rarely reported with few case reports [10–12]. Javelot et al. in 2015 described reflux esophagitis associated with clozapine use in a 58-year-old chronic schizophrenic patient with hiatal hernia [11]. Laker and Cookson in 1997 reported erosive esophagitis in patients within 6 weeks of starting clozapine [12]. As seen in both cases, clozapine may be related to our patient's development of esophagitis, given the timing and no other identifiable plausible triggering events.

Esophagitis can have multiple etiologies, including various caustic chemical agents, infections, ischemic events, pill-induced, and reflux. Given the patient was in the controlled setting, ingestion of unknown caustic substance was highly unlikely. Her mild leukocytosis upon transfer to medicine service was likely due to esophageal inflammation and less likely infectious, given the tissue biopsy did not show evidence of local esophageal infection. The tissue biopsy showed necrotic components of the inflamed mucosa; however, she did not exhibit systemic inflammatory conditions or significant hemodynamic compromise that may have caused the hypoperfusion of esophageal tissue.

Pill esophagitis is another potential type of esophagitis. Although this usually occurs in the midupper esophagus due to indentations caused by the surrounding anatomy, in this patient's case, the distal ulcerations may have occurred due to the hypomobility of the esophagus caused by clozapine's anticholinergic properties [13]. In addition, the anticholinergic effect may reduce the lower esophageal sphincter pressure, exacerbating the reflux of the gastric content [12]. Given the presence of hiatal hernia, our patient may have had undiagnosed gastroesophageal reflux disease, although endoscopic findings of punctate ulcerations are atypical of reflux esophagitis.

Based on the clinical course, the likely triggering event in our patient was the initiation of clozapine. The underlying mechanism may be driven by clozapine's highly antimuscarinic property, by causing esophageal dysmotility and reducing the lower esophageal sphincter pressure. With supportive management (pantoprazole), our patient had a clinical improvement of gastrointestinal symptoms while she continued to be on clozapine and even when the dosage of clozapine was further increased.

We present a case of a patient who developed coffee-ground emesis secondary to severe esophagitis 14 days after the initiation of clozapine. The clinical evaluation proved challenging due to the difficulty with assessment of the patient's symptoms. We hypothesize that esophagitis may be a side effect of clozapine, an event that warrants further attention. We observed that pantoprazole helped with resolution of gastrointestinal symptoms, and therefore, proton pump inhibitors may have a role in preventing esophagitis in patients taking clozapine. Further research is needed to elucidate whether there is a direct causative link between clozapine and esophagitis and the potential underlying mechanism.
